# Supplementary material for: The Peripheral Immune Traits Changed in Patients with Multiple System Atrophy
Source: Brain Sci. 2023 Jan 26;13(2):205. doi: 10.3390/brainsci13020205 (PMC9953988; doi:10.3390/brainsci13020205)
Supplement: Supplementary file 1 [file brainsci-13-00205-s001.zip › brainsci-2120275-Table S1.pdf]

Supplementary Table S1. Spearman correlations of clinical characteristics with lymphocyte subsets of MSA patients.

Indices	Age at onset		Disease duration	
	R	*P*	R	*P*
**Lymphocyte Subsets**				
Total T cells ($CD3^{+}CD19^{-}$) (%)	−0.375	0.074	0.070	0.736
Total T cell count ($CD3^{+}CD19^{-}$) (/μL)	−0.108	0.600	−0.030	0.885
Total B cells ($CD3^{-}CD19^{+}$) (%)	−0.277	0.170	0.054	0.794
Total B cell count ($CD3^{-}CD19^{+}$) (/μL)	−0.201	0.325	−0.001	0.997
T/B ($\times 10^{9}$/L)	0.144	0.484	−0.025	0.904
NK cells ($CD3^{-}CD16^{+}CD56^{+}$) (%)	0.463	**0.017**	−0.126	0.539
NK cell count ($CD3^{-}CD16^{+}CD56^{+}$) (/μL)	0.427	**0.030**	−0.111	0.588
T+B+NK (%)	−0.361	0.070	0.093	0.653
T+B+NK cell count (/μL)	−0.057	0.784	−0.048	0.817
**T cells Subsets**				
Th cells ($CD3^{+}CD4^{+}$) (%)	−0.036	0.860	−0.142	0.488
Th cell count ($CD3^{+}CD4^{+}$) (/μL)	−0.074	0.718	−0.152	0.460
Tc cells ($CD3^{+}CD8^{+}$) (%)	−0.248	0.223	0.282	0.162
Tc cell count ($CD3^{+}CD8^{+}$) (/μL)	−0.300	0.137	0.356	0.075
Th/Tc	0.171	0.403	−0.311	0.122
Th ($CD3^{+}CD4^{+}CD28^{+}$)/Th (%)	−0.274	0.176	−0.286	0.156
Tc ($CD3^{+}CD8^{+}CD28^{+}$)/Tc (%)	−0.264	0.192	−0.372	0.061
Activated T cells ($CD3^{+}HLA\text{-}DR^{+}$) (%)	0.116	0.573	0.343	0.086
Activated Tc cells ($CD3^{+}CD8^{+}HLA\text{-}DR^{+}$)/Tc (%)	0.462	**0.017**	0.349	0.080
Treg (%) ($CD3^{+}CD4^{+}CD25^{+}CD127low^{+}$)	0.119	0.563	0.093	0.650
Natural Treg (%) ($CD45RA^{+}CD3^{+}CD4^{+}CD25^{+}CD127low^{+}$)	−0.097	0.636	0.109	0.598
Induced Treg (%) ($CD45RO^{+}CD3^{+}CD4^{+}CD25^{+}CD127low^{+}$)	0.164	0.422	0.057	0.784
Induced Treg/Natural Treg	−0.259	0.202	−0.046	0.822

Abbreviations: HC, healthy controls; MSA, multiple system atrophy; NK cell, natural killer cell; Tc cell, cytotoxic T cell; Th cell, helper T cell; Treg, regulatory T cell.
