# Supplementary material for: The Peripheral Immune Traits Changed in Patients with Multiple System Atrophy
Source: Brain Sci. 2023 Jan 26;13(2):205. doi: 10.3390/brainsci13020205 (PMC9953988; doi:10.3390/brainsci13020205)
Supplement: Supplementary file 1 [file brainsci-13-00205-s001.zip › brainsci-2120275-Table S2.pdf]

Supplementary Table S2. Comparison of lymphocyte subsets data in MSA patients of different genders.

Indices	Participants		Effective size	*P*-value
	Male (*N* = 16)	Female (*N* = 10)		
**Lymphocyte Subsets**				
Total T cells ($CD3^{+}CD19^{-}$) (%)	68.35 (61.17,75.37)	74.21 (69.47,75.58)	−1.423	0.155[b]
Total T cell count ($CD3^{+}CD19^{-}$) (/μL)	1201.69 (332.81)	1158.60 (184.23)	0.424	0.675[a]
Total B cells ($CD3^{-}CD19^{+}$) (%)	12.58 (4.76)	12.90 (3.35)	−0.185	0.855[a]
Total B cell count ($CD3^{-}CD19^{+}$) (/μL)	228.13 (116.36)	210.30 (75.52)	0.474	0.640[a]
T/B (×10^{9}/L)	6.03 (3.79,7.29)	5.85 (4.25,7.90)	−0.211	0.833[b]
NK cells ($CD3^{-}CD16^{+}CD56^{+}$) (%)	18.18 (8.09)	14.09 (3.20)	1.809	0.085[a]
NK cell count ($CD3^{-}CD16^{+}CD56^{+}$) (/μL)	325.38 (187.74)	225.10 (59.71)	1.982	0.062[a]
T+B+NK (%)	99.38 (98.90,99.60)	99.34 (99.08,99.58)	−0.264	0.792[b]
T+B+NK cell count (/μL)	1572.50 (1346.75,2261.25)	1512.50 (1418.25,1864.00)	−0.264	0.792[b]
**T cells Subsets**				
Th cells ($CD3^{+}CD4^{+}$) (%)	45.85 (6.22)	46.56 (5.94)	−0.286	0.777[a]
Th cell count ($CD3^{+}CD4^{+}$) (/μL)	817.44 (271.60)	745.7 (148.45)	0.869	0.394[a]
Tc cells ($CD3^{+}CD8^{+}$) (%)	20.18 (6.72)	22.11 (5.97)	−0.742	0.466[a]
Tc cell count ($CD3^{+}CD8^{+}$) (/μL)	340.88 (110.58)	353.50 (99.66)	−0.294	0.771[a]
Th/Tc	2.29 (1.64,3.41)	2.05 (1.81,3.11)	−0.369	0.712[b]
Th ($CD3^{+}CD4^{+}CD28^{+}$)/Th (%)	98.15 (95.16,99.11)	96.76 (91.10,98.15)	−1.370	0.171[b]
Tc ($CD3^{+}CD8^{+}CD28^{+}$)/Tc (%)	62.97 (17.94)	63.80 (9.88)	−0.151	0.881[a]
Activated T cells ($CD3^{+}$HLA-DR^{+}) (%)	14.75 (6.31)	14.31 (2.95)	0.240	0.813[a]
Activated Tc cells ($CD3^{+}CD8^{+}$HLA-DR^{+})/Tc (%)	36.32 (13.81)	37.65 (9.39)	−0.292	0.773[a]
Treg (%) ($CD3^{+}CD4^{+}CD25^{+}CD127low^{+}$)	3.93 (1.37)	3.55 (1.09)	0.742	0.466[a]
Natural Treg (%) ($CD45RA^{+}CD3^{+}CD4^{+}CD25^{+}CD127low^{+}$)	0.99 (0.49)	0.87 (0.36)	0.655	0.519[a]
Induced Treg (%) ($CD45RO^{+}CD3^{+}CD4^{+}CD25^{+}CD127low^{+}$)	2.80 (2.03,4.01)	2.94 (1.76,3.51)	−0.659	0.510[b]
Induced Treg/Natural Treg	0.36 (0.18)	0.34 (0.16)	0.232	0.818[a]

Data presented as mean (SD) or median (25% quantile, 75% quantile). P value calculated by Student's t-test[a] or Mann-Whitney U-test[b]. Abbreviations: MSA, multiple system atrophy; Th cell, helper T cell; Tc cell, cytotoxic T cell; NK cell, natural killer cell; Treg, regulatory T cell; SD, standard deviation.
